# Platelets as Potential Non-Traditional Cardiovascular Risk Factor—Analysis Performed in Healthy Donors

**DOI:** 10.3390/ijms241914914

**Published:** 2023-10-05

**Authors:** Patrycja Szymańska, Bogusława Luzak, Przemysław Siarkiewicz, Jacek Golański

**Affiliations:** 1 Department of Haemostasis and Haemostatic Disorders, Chair of Biomedical Sciences, Medical University of Lodz, Mazowiecka 6/8, 92-215 Lodz, Poland; patrycja.szymanska@umed.lodz.pl (P.S.); boguslawa.luzak@umed.lodz.pl (B.L.); 2 Institute of Polymer and Dye Technology, Faculty of Chemistry, Lodz University of Technology, Stefanowskiego 16, 90-537 Lodz, Poland; przemyslaw.siarkiewicz@p.lodz.pl

**Keywords:** platelets, cardiovascular disease, non-traditional risk factor, cardiometabolic risk factors, dyslipidemia, hyperglycemia, overweight, platelet reactivity, reactive oxygen species, inflammatory markers

## Abstract

Abnormal lipid profile, increased glucose level, and elevated body weight are traditional cardiometabolic risk factors; however, the role of platelets in the development of cardiovascular disease (CVD) is increasingly being highlighted. The aim of this study was to select platelet-related parameters (non-genetic molecular and routine laboratory measurements) that may be associated with increased cardiovascular risk among healthy populations. We evaluated the level of platelet indices, platelet-based inflammatory markers, platelet reactivity parameters, and platelet reactive oxygen species (ROS) generation in relation to selected cardiometabolic risk factors. We noted the association between total cholesterol and LDL cholesterol with platelet aggregation and platelet ROS generation. We found the relationship between triglycerides, glucose, and body mass index with the relatively new multi-inflammatory indices (MII-1 and MII-3). Moreover, we noticed that the mean platelet volume-to-lymphocyte ratio in healthy subjects is not a good source of information about platelets and inflammation. We also highlighted that platelet-to-HDL-cholesterol ratio may be a promising prognostic cardiometabolic indicator. The association between platelet-related (especially molecular) and cardiometabolic parameters requires further research. However, the goal of this study was to shed light on the consideration of platelets as a non-traditional cardiovascular risk factor and a crucial element in identifying individuals at high-risk of developing CVD in the future.

## 1. Introduction

Cardiovascular diseases (CVD) are non-communicable disorders responsible for a huge number of deaths worldwide. Among the traditional CVD risk factors, we can point out genetic, environmental, lifestyle, and clinical [1,2]. However, it is also noteworthy to highlight platelets as a potential non-traditional risk factor of CVD. Platelets are mainly involved in hemostasis and thrombosis. Activated platelets secrete various substances that affect not only physiological but also pathological processes, including inflammation. Their role depends on lipid metabolism, which can affect platelet activation and aggregation, and contribute to thrombin generation and thrombus formation [3].

Atherosclerosis is a chronic inflammatory vascular disease in which platelets play a crucial role [4,5]. The development of atherosclerosis, with associated inflammation, is dependent on platelet activation and aggregation [6]. In addition, metabolic disorders contribute to the progression of this condition [7,8], as well as to platelet dysfunction. The pro-thrombotic tendency is also influenced by oxidative stress and chronic low-grade inflammation [9,10].

Dyslipidemia is associated with an increased risk of adverse cardiovascular incidents. Elevated cholesterol level, sustained for a long time, increases platelet reactivity [4,11]. Platelets in individuals with elevated cholesterol levels are characterized by increased aggregation, expression of P-selectin, and the fibrinogen binding, as well as increased generation of superoxide anion [10,12]. Nkambule et al. noted the effect of hypertriglyceridemia on platelet aggregation and activation. They also pointed out that an increase in thrombotic susceptibility is directly related to high triglyceride levels [13]. Navarese et al. indicated an association between a reduction of all-cause and cardiovascular mortality and a decrease in low-density lipoprotein (LDL) cholesterol level when the baseline level was above 100 mg/dL [14]. Statins, which are commonly used to treat lipid disorders, not only reduce LDL cholesterol levels but also platelet reactivity [15]. The development of atherosclerotic lesions can also be exacerbated by elevated glucose levels [16].

The increased reactive oxygen species (ROS) production can lead to oxidative stress [17]. It may occur as a result of elevated levels of free fatty acids and glucose. Increased ROS generation has also been linked to atherosclerosis [18] and prothrombotic state [19]. ROS also affect the regulation of platelet function [20]. Hypercholesterolemia through the interaction of platelet-activated membrane with oxidized LDL (oxLDL) is associated with platelet hyperreactivity and predisposition to thrombus formation. Monitoring platelet oxidative stress biomarkers may be beneficial in the identification of patients with prothrombotic tendencies and increased cardiovascular risk [19].

CD40 ligand (CD40L), a molecular marker, is present on activated platelets and plays a key role in thrombosis and inflammation [21]. Elevated CD40L may be noted among those with hypercholesterolemia [10,12], and assessment of CD40L level can be considered a platelet-based indicator of cardiovascular risk [21,22].

C-reactive protein (CRP) may reflect inflammation occurring in the progression of metabolic conditions, such as diabetes. CRP can also predict the risk of adverse cardiovascular events, as well as cardiac death [23,24]. Assessment of CRP levels and lipid profile may be helpful in identifying individuals at high-risk for CVD and may be an important component of primary CVD prevention [25].

Inflammatory markers, which are calculated based on complete blood count, combine information about inflammation and thrombosis. Elevated platelet-to-lymphocyte ratio (PLR) is associated with platelet activation, inflammation, and atherosclerosis. This indicator can be a prognostic marker for many cardiovascular events [26] and all-cause mortality among patients with acute coronary syndrome (ACS) [27]. Mean platelet volume (MPV)-to-lymphocyte ratio (MPVLR) may be a prognostic indicator of thrombus formation in patients with ST-segment elevation myocardial infarction (STEMI) [28] and a predictor of mortality among patients after acute cardiovascular incidents [29]. The systemic immune-inflammation index (SII) may be a better predictor of adverse cardiovascular events than traditional risk factors [30]. In the study by Ye et al., the SII level was associated with an increased risk of CVD [31]. Xia et al. reported a relationship between SII and death from cardiovascular events, as well as from all-cause mortality [32]. Mahemuti et al. found that an increase in SII value correlates with hyperlipidemia [33]. The aggregate index of systemic inflammation (AISI) is a new inflammatory marker that includes the platelet, neutrophile, and lymphocyte count (similar to SII), as well as monocyte count. In the study by Song et al., AISI level was higher among patients with type 2 diabetes and peripheral artery disease and was also associated with disease severity [34]. Using this marker can help identify individuals at high-risk of CVD. A relatively new index associated with systemic inflammation is the multi-inflammatory index (MII): MII-1 (PLR × CRP) and MII-3 (SII × CRP). Doğanay et al. reported increased MII levels in patients after percutaneous coronary intervention (PCI) due to ACS. MII-3 had greater prognostic efficacy regarding the risk of death from acute thrombosis after coronary intervention and can be used to identify patients at increased risk of postoperative complications at the qualification stage for PCI [35].

We consider that platelets may play a crucial role in identifying individuals at high cardiovascular risk, which can subsequently lead to the development of CVD. Since there are many studies on metabolic parameters and platelet function performed in patients with CVD, in our study, we decided to focus on a group of healthy individuals without diagnosed chronic diseases. The main goal of our study was to attempt to identify parameters related to platelet function and inflammatory markers associated with platelet count (classified as non-genetic molecular or routine laboratory measurements) that may be crucial in assessing cardiovascular risk among healthy adults. We hypothesize that platelet-based indicators may be potential non-traditional predictors of CVD; thus, inclusion of some of them in the assessment of cardiovascular risk may expand the panel of current medical tests, support the screening, and contribute to more effective primary CVD prevention.

## 2. Results

### 2.1. General Characteristics of Participants

A total of 94 donors, including 49 women (52%) and 45 men (48%) with a mean age of 39.6 ± 15.3 years, participated in the study. Basic characteristics of blood morphology, biochemical, inflammatory, and platelet function parameters are presented in Table 1. Other parameters describing the donors are shown in Table S1 in the Supplementary Materials. Donors participating in the study had declared the absence of chronic metabolic disorders, such as dyslipidemia or diabetes, and inflammatory diseases as diagnosed up to the date of the examination. They also declared the absence of taking antiplatelet or anticoagulant drugs.

To assess the association between platelet parameters, platelet-based inflammatory markers, platelet reactivity indices, or platelet ROS generation and cardiovascular risk indicators (total cholesterol (TC), LDL cholesterol (LDL-C), non-HDL cholesterol (non-HDL-C), triglycerides (TG), glucose, and body mass index (BMI)), donors were divided into two subgroups (based on the median), with lower or higher levels of the parameters described above as cardiovascular risk factors. The cut-off values were as follows: TC—5.23 mmol/L; LDL-C—3.13 mmol/L; non-HDL-C—3.74 mmol/L; TG—1.12 mmol/L; glucose—5.25 mmol/L; and BMI—24.3 kg/m^2^.

### 2.2. General Characteristics of Platelet Parameters, Platelet-Based Inflammatory Markers, Platelet Reactivity Parameters, and Platelet Reactive Oxygen Species Generation

We analyzed the level of platelet indices, such as platelet count (PLT), mean platelet volume (MPV), platelet crit (PCT), platelet distribution width (PDW), and platelet mass index (PMI), as well as platelet-based inflammatory markers such as PLR (platelet/lymphocyte ratio), SII (systemic immune-inflammation index), AISI (aggregate index of systemic inflammation), MII-1 (multi-inflammatory index-1), and MII-3 (multi-inflammatory index-3). Moreover, we also made a comparative analysis of parameters describing platelet reactivity: collagen-induced platelet aggregation, P-selectin expression on collagen-activated platelets, fibrinogen binding to collagen-activated platelets (at two incubation times: at rest and after 60 min at 37 °C), and the platelet ROS generation induced with collagen or thrombin (stimulation with each agonist for 5 and 10 min). We also determined plasma levels of interleukin-6 (IL-6) and CD40 ligand (CD40L) and calculated two additional parameters: platelet/HDL cholesterol ratio (PHR) and MPV/lymphocyte ratio (MPVLR). Then, we compared the level of the above-mentioned parameters in relation to the level of cardiovascular risk factors (subgroup I—lower than median level; subgroup II—higher than median level).

**Figure 1:**
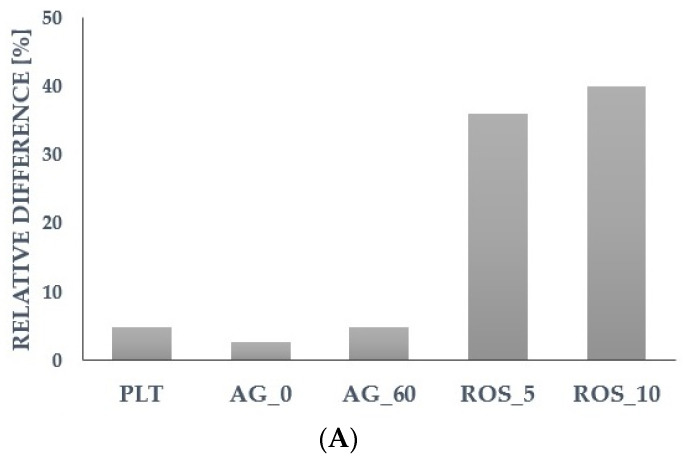

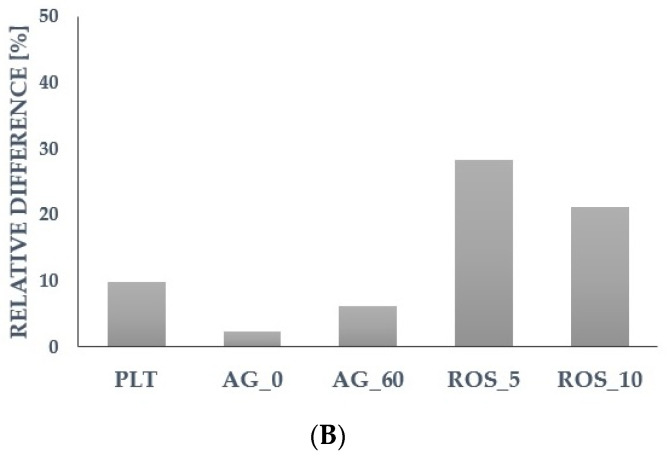
Differences in selected platelet-associated parameters in relation to (**A**)—total cholesterol; and (**B**)—LDL cholesterol. Data are presented as relative difference expressed as percentages (calculated based on the median in the group with higher level vs. lower level of total cholesterol or LDL cholesterol, respectively). PLT—platelet count; AG_0—collagen-induced aggregation in platelets at rest; AG_60—collagen-induced aggregation in platelets after 60 min; ROS_5—reactive oxygen species generation in collagen-stimulated platelets for 5 min; ROS_10—reactive oxygen species generation in collagen-stimulated platelets for 10 min.

**Figure 2:**
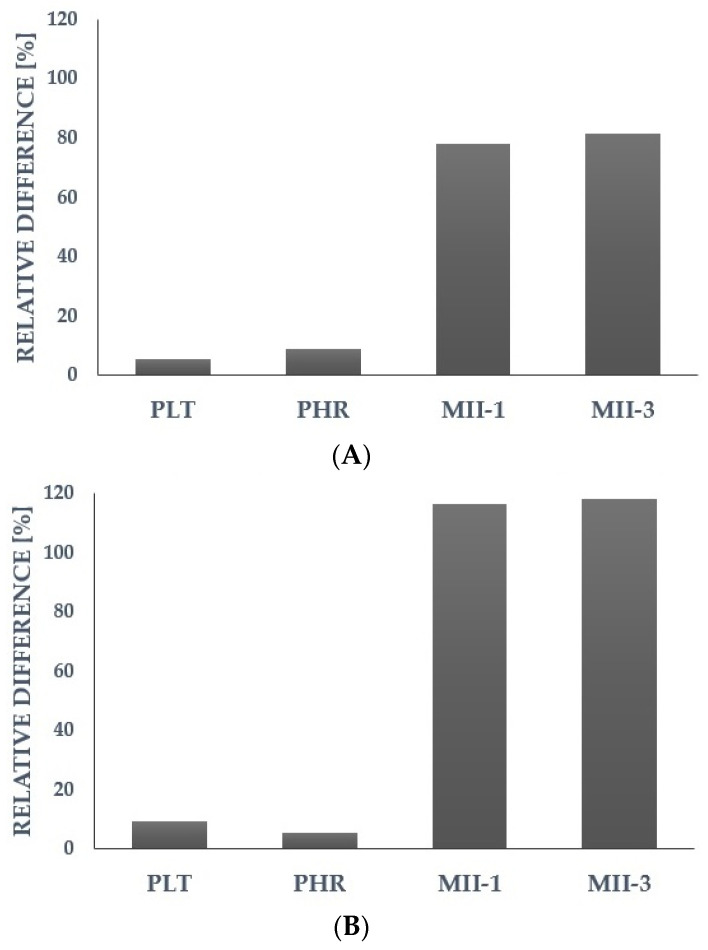

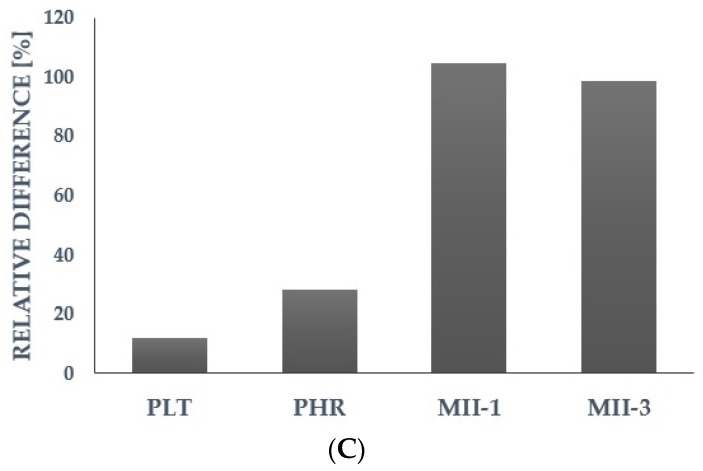
Differences in selected platelet-associated parameters in relation to (**A**)—triglycerides; (**B**)—glucose; and (**C**)—body mass index. Data are presented as relative difference expressed as percentages (calculated based on the median in the group with higher level vs. lower level of triglycerides or glucose, or body mass index, respectively). PLT—platelet count; PHR—platelet/HDL cholesterol ratio; MII-1—multi-inflammatory index-1 (PLR × CRP); MII-3—multi-inflammatory index-3 (SII × CRP).

For further analysis, we selected the most promising parameters that showed a predictive potential for cardiovascular risk factors during the assessment of the relative differences (calculated based on the median) in the group with a higher level vs. the group with a lower level of a particular cardiometabolic parameter (Figure 1 and Figure 2). We assumed that for the higher levels of risk factors (TC, LDL-C, TG, glucose, BMI), the platelet reactivity, platelet ROS generation, or platelet-related inflammatory markers might also be higher. The detailed results of other parameters were presented in the Supplementary Materials (Tables S2 and S3).

### 2.3. Association between Cardiovascular Risk Factors and Platelet Parameters, Platelet-Based Inflammatory Markers, Platelet Reactivity Parameters, and Platelet Reactive Oxygen Species Generation

Below, we report the characteristics of platelet count and platelet function parameters, including reactivity based on measurements of optical aggregation in collagen-induced platelets, as well as the ROS generation in collagen-stimulated platelets in relation to TC and LDL-C levels (Table 2).

Among all the platelet parameters, platelet count was statistically significantly different for LDL-C (*p* = 0.026). The subgroup with higher LDL-C (Me = 3.73 mmol/L; IQR: 3.47–4.28 mmol/L) had a higher platelet count than the subgroup with lower LDL-C (Me = 2.56 mmol/L, IQR: 2.23–2.88 mmol/L). We found no correlation between platelet count and TC or LDL-C level. In addition, we noted statistically significant differences for collagen-induced aggregation in platelets after 60 min at 37 °C in the TC (*p* = 0.040) and LDL-C (*p* = 0.022) subgroups. The subgroup with higher TC (Me = 5.91 mmol/L; IQR: 5.41–6.56 mmol/L) and higher LDL-C had increased collagen-induced aggregation in platelets after 1 h at 37 °C compared to the subgroup with lower TC (Me = 4.43 mmol/L; IQR: 4.05–4.98 mmol/L) and LDL-C, respectively. We found a positive correlation between TC level and collagen-induced aggregation in platelets after 1 h at 37 °C (*r* = 0.240, *p* = 0.037), while between this platelet reactivity parameter and LDL-C, there was only a statistical tendency for significance (*r* = 0.222, *p* = 0.055). Statistically significant differences were found between TC subgroups for ROS generation in platelets stimulated with collagen for 5 min (*p* = 0.031) and 10 min (*p* = 0.040). The subgroup with higher TC had elevated collagen-induced ROS generation for 5 min and 10 min compared to the subgroup with lower levels of TC. There were no statistically significant correlations between TC and ROS generation in collagen-stimulated platelets. However, there was a statistical tendency for significance between LDL-C and collagen-induced ROS generation for 5 min (*r* = 0.224, *p* = 0.057) and 10 min (*r* = 0.221, *p* = 0.060).

In Table 3 we presented the characteristics of platelet count and other parameters related to platelets, such as PHR, MII-1, and MII-3 in relation to TG, glucose, and BMI.

Platelet count was statistically significantly different between glucose (*p* = 0.047) subgroups, while for BMI, it remained at the limit of statistical significance (*p* = 0.059). The subgroup with higher glucose (Me = 5.61 mmol/L; IQR: 5.36–5.79 mmol/L) and higher BMI (Me = 27.2 kg/m^2^; IQR: 26.3–29.3 kg/m^2^) had a higher platelet count than the subgroup with lower glucose (Me = 4.98 mmol/L; IQR: 4.77–5.14 mmol/L) and lower BMI (Me = 21.9 kg/m^2^; IQR: 20.6–22.9 kg/m^2^), respectively. We found no correlation between platelet count and TG, glucose, or BMI. MII-1 and MII-3 were statistically significantly different for TG (*p* = 0.002 and *p* = 0.001), glucose (*p* < 0.001 and *p* < 0.001), and BMI (*p* = 0.005 and *p* = 0.002). The subgroup with higher TG (Me = 1.43 mmol/L; IQR: 1.23–1.98 mmol/L), higher glucose, and higher BMI had increased MII-1 and MII-3 values compared to the subgroup with lower TG (Me = 0.81 mmol/L; IQR: 0.70–1.00 mmol/L), glucose, and BMI. We found a positive correlation only between two inflammatory markers, MII-1 and MII-3, and TG (*r* = 0.377, *p* < 0.001 and *r* = 0.430, *p* < 0.001), glucose (*r* = 0.368, *p* < 0.001 and *r* = 0.359, *p* < 0.001), and BMI (*r* = 0.357, *p* = 0.002 and *r* = 0.369, *p* = 0.002), respectively. PHR was statistically significantly different for glucose (*p* = 0.041) and BMI (*p* = 0.001). The subgroup with higher glucose and higher BMI had elevated PHR values compared to the subgroup with lower glucose and BMI. We noted a positive correlation between PHR and TG (*r* = 0.251, *p* = 0.015) and BMI (*r* = 0.381, *p* = 0.001). There was also a statistical tendency for significance between PHR and glucose (*r* = 0.197, *p* = 0.057).

### 2.4. Influence of Age and Gender on Associations between Cardiovascular Risk Factors and Platelet Parameters, Platelet-Based Inflammatory Markers, Platelet Reactivity Parameters, and Platelet Reactive Oxygen Species Generation

We also decided to evaluate the correlation between the above-analyzed platelet-related and cardiometabolic parameters according to age (younger vs. older donors; median–cut-off value: 36 years) and gender (females vs. males).

Among platelet parameters, we noted a positive correlation between platelet count and TC (*r* = 0.316, *p* = 0.035) and LDL-C (*r* = 0.439, *p* = 0.003) in the male group. Collagen-induced aggregation in platelets at rest was also positively correlated with TC (*r* = 0.401, *p* = 0.014) and LDL-C (*r* = 0.412, *p* = 0.013) in the male group. In contrast, collagen-induced aggregation in platelets after 60 min at 37 °C was correlated in the female group with TC (*r* = 0.357, *p* = 0.022). Additionally, in the older donor group, there was a positive correlation between collagen-induced aggregation in platelets at rest and TC (*r* = 0.353, *p* = 0.037). Generation of collagen-induced ROS for 5 and 10 min was positively correlated with TC (*r* = 0.395, *p* = 0.013 and *r* = 0.346, *p* = 0.031) and LDL-C (*r* = 0.399, *p* = 0.013 and *r* = 0.337, *p* = 0.038) only in the younger donor group.

Moreover, in the female group, platelet count was positively correlated with TG (*r* = 0.345, *p* = 0.015) and BMI (*r* = 0.412, *p* = 0.008). Regarding inflammatory markers, we found a positive correlation in the female group between MII-1 and MII-3 and TG (*r* = 0.375, *p* = 0.008 and *r* = 0.426, p = 0.002), glucose (*r* = 0.487, *p* < 0.001 and *r* = 0.484, *p* < 0.001), and BMI (*r* = 0.427, *p* = 0.006 and *r* = 0.381, *p* = 0.015). In the male group, MII-1 and MII-3 were correlated positively with BMI (*r* = 0.368, *p* = 0.042 and *r* = 0.400, *p* = 0.026), and for TG, the correlation was about the limit of statistical significance (*r* = 0.290, *p* = 0.056 and *r* = 0.300, *p* = 0.048). Additionally, in the younger donors, MII-1 and MII-3 were positively correlated with TG (*r* = 0.378, *p* = 0.008 and *r* = 0.443, *p* = 0.002) and BMI (*r* = 0.371, *p* = 0.022 and *r* = 0.373, *p* = 0. 021), and in the older donors with TG (*r* = 0.344, *p* = 0.021 and *r* = 0.382, *p* = 0.010), but also with glucose (*r* = 0.670, *p* < 0.001 and *r* = 0.651, *p* < 0.001). In contrast, correlations between MII-1 and MII-3 and BMI in the older donor group remained at the limit of statistical significance (*r* = 0.344, *p* = 0.058 and *r* = 0.344, *p* = 0.050). PHR was positively correlated with TG (*r* = 0.346, *p* = 0.015), glucose (*r* = 0.342, *p* = 0.016), and BMI (*r* = 0.423, *p* = 0.007) in the female group, while in the male group, only with BMI (*r* = 0.370, *p* = 0.040). We found a positive correlation between PHR and TG (*r* = 0.334, *p* = 0.020) and BMI (*r* = 0.377, *p* = 0.020) in the younger donors, while in the older donors for glucose (*r* = 0.324, *p* = 0.028).

### 2.5. Potential Predictors of Cardiometabolic Risk Factors Levels

After analyzing simple correlations between platelet and cardiometabolic parameters in relation to age and gender, in the next step, we performed multiple regression analysis to attempt the prediction of selected cardiometabolic parameters’ levels due to potential predictors, such as platelet count, inflammatory markers, and platelet function parameters. We included the unadjusted model and models adjusted for age, for gender, and for both age and gender.

In Table 4, the effects of variables such as platelet count, aggregation, and ROS generation in collagen-stimulated platelets on TC and LDL-C levels were evaluated. No significant association between TC and LDL-C and ROS generation for 5 and 10 min was found either in the unadjusted model or in all adjusted models. We also noted no significant association between TC and LDL-C and platelet count and aggregation in platelets at rest and after 1 h at 37 °C in the unadjusted model. In contrast, in the age-adjusted model, we found a significant association for TC or LDL-C and aggregation in platelets at rest and after 1 h at 37 °C, while in the gender-adjusted model, there was a significant association for TC and LDL-C not only for platelet aggregation but also for platelet count. In contrast, after adjusting the model for age and gender together, the association had changed and became stronger for both aggregations, but not for platelet count.

In Table 5, the effects of variables such as platelet count, PHR, MII-1, and MII-3 on TG, glucose, and BMI were evaluated. The multiple regression in the unadjusted model confirms that PHR, MII-1, and MII-3 were positively associated with TG, glucose, and BMI. After adjustment for age or gender, or age and gender together, the association between the analyzed parameters remained remarkable but became weaker. In contrast, platelet count was associated with BMI only in models adjusted for gender or for age and gender.

### 2.6. Association between Cardiovascular Risk Factors and Mean Platelet Volume (MPV), and MPV to Lymphocyte Ratio (MPVLR)

In addition, we also analyzed the association between selected cardiovascular risk factors and other parameters potentially related to inflammation or/and platelet activation, such as mean platelet volume (MPV) and MPV/lymphocyte ratio (MPVLR).

Interestingly, we noted statistically significant negative correlations between MPV or MPVLR and most cardiometabolic parameters, as well as between MPV or MPVLR and platelet count (Table 6). Moreover, statistically significantly higher MPVLR levels were noted in the subgroup with lower TC (*p* = 0.002), LDL-C (*p* = 0.006), non-HDL-C (*p* = 0.006), and TG (*p* = 0.024) compared to the subgroups with higher levels of the aforementioned parameters (Table S2 in Supplementary Materials).

## 3. Discussion

The presence of hyperlipidemia, diabetes, and overweight is undeniably linked to higher cardiovascular risk. Elevated levels of lipids, glucose, and body weight are traditional cardiometabolic risk factors that, if inadequately monitored and treated, lead to the development of CVD [2]. In our study, we recruited potentially healthy donors who did not have a history of chronic metabolic or inflammatory disease. However, after analyzing our donors’ laboratory results, many of them had abnormal levels of metabolic parameters. We noted elevated TC levels (>5 mmol/L) in 61% of donors (*n* = 57), LDL-C (>3 mmol/L) in 57% of individuals (*n* = 54), and non-HDL-C (>3.8 mmol/L) in 46% of subjects (*n* = 43). Abnormal TG levels (>1.7 mmol/L) were found in 15% of donors (*n* = 14). Furthermore, increased glucose levels (>5.5 mmol/L) were detected in 29% of participants (*n* = 27). Moreover, 36% of donors (*n* = 34) were found to be overweight (BMI >24.9 kg/m^2^). Only HDL-C levels did not deviate from normal levels in all study group (*n* = 94) among both women (>1.2 mmol/L) and men (>1 mmol/L).

The role of platelets in the development of CVD is increasingly being highlighted, and effective therapy for such conditions involves antiplatelet drugs [36]. Therefore, we consider that platelets can be regarded as a non-traditional CVD risk factor. The aim of our study was to compare platelet parameters and platelet function in potentially healthy populations which were divided into subgroups based on the median values of particular cardiometabolic parameters, known to be CVD risk factors.

In this study, we found that platelet reactivity based on collagen-stimulated platelet aggregation (platelets after 1 h at 37 °C) was statistically significantly higher in the subgroup with higher TC (*p* = 0.040), as well as in the subgroup with higher LDL-C (*p* = 0.022), compared to the subgroups with lower TC and LDL-C levels, respectively. Referring to the literature, increased platelet activation is associated with the occurrence of hyperlipidemia. It has been noted that the state of hypercholesterolemia has a greater impact on platelet activation compared to hypertriglyceridemia. Excessive platelet activation and the following increased reactivity, associated with lipid disorders, increase the risk of atherosclerosis, as well as thrombotic incidents [11]. Ex vivo studies of platelet function confirm the link between increased platelet activation and lipid disorders, as well as adverse cardiovascular events among patients with coronary artery disease [37]. In vitro study by Betteridge et al. reported increased platelet reactivity among patients with familial hypercholesterolemia or coronary artery disease in response to platelet stimulation with traditional agonists [38]. In a study by Singh et al., platelet aggregation was statistically significantly elevated in patients with hyperlipidemia compared to controls (*p* < 0.005) [39].

From the group of non-genetic molecular indicators, we noted a statistically significant difference in ROS generation in platelets stimulated with collagen for 5 min (*p* = 0.031) and for 10 min (*p* = 0.040) in the subgroup with higher TC compared to the subgroup with lower TC. A study by Qiao et al. highlighted the relationship between platelet function and ROS generation and reported that intracellular ROS production in human platelets can occur through ligand binding with the platelet collagen receptor—glycoprotein VI [40]. Previous experimental studies have confirmed the association between increased production of superoxide anion and the occurrence of elevated cholesterol levels. A study by Sanguigni et al. reported significantly higher superoxide anion production in platelets from patients with hypercholesterolemia (*p* < 0.001) compared to controls. In addition, it was statistically significantly associated with LDL-C levels (*p* < 0.001) [41]. The association of ROS generation with traditional risk factors is noteworthy. In our study, we used a relatively simple and cost-effective method to determine ROS generation, and we believe that it is worthy of recommendation for further research.

In summary, we reported the relationship between lipid parameters and platelet aggregation or ROS generation in collagen-stimulated platelets. In contrast, we did not find any association for several promising molecular parameters, such as CD40L and platelet reactivity assessed by fibrinogen binding and P-selectin expression involving collagen-induced platelets. These results suggest that in mild hypercholesterolemia, collagen-induced platelet aggregation seems to be a more sensitive parameter of platelet reactivity than other markers, especially those related to the early phase of platelet activation.

In the analysis of the platelet parameters, we noted a statistically significant elevated platelet count (*p* = 0.026) among individuals with higher LDL-C compared to the group with a lower level of this parameter. In dyslipidemic conditions, hematopoiesis may be disrupted, thereby leading to increased platelet production and induction of a prothrombotic phenotype [37].

Analyzing inflammatory markers, we found a positive correlation between BMI and MII-1, as well as between BMI and MII-3. Obesity and inflammation are closely related and, together, they are significant risk factors for the development of CVD. Inflammation associated with excess body weight, resulting in overweight or obesity, induces chronic low-grade inflammation, which leads to an increased risk of metabolic disorders and CVD. Therefore, as obesity is regarded as a pro-inflammatory condition, which results in increased cardiovascular risk, reducing excess body weight seems to be crucial in decreasing inflammatory processes and CVD prevalence [42].

In our study, TG level was also statistically significantly correlated with inflammatory markers: MII-1 and MII-3. There are reports suggesting that the presence of hypertriglyceridemia predisposes much more to low-grade inflammation than elevated LDL-C [43]. A study by Okada et al. showed that elevated TG were associated with high-sensitivity CRP (hs-CRP), regardless of LDL-C or BMI, so it can be inferred that this inflammatory marker may inform us of inflammation due to an elevated TG level [44]. Preclinical and clinical studies are increasingly focusing on the impact of elevated TG on the development of atherosclerosis-induced CVD, and the major role of inflammation in this process. It has been suggested that future CVD therapies should focus on reducing hypertriglyceridemia and inflammation [43].

Furthermore, we also reported statistically significant positive correlations between inflammatory parameters, MII-1 and MII-3, and glucose. Elevated glucose level, which predisposes to the development of type 2 diabetes, is also associated with chronic low-grade systemic inflammation [45]. Pitsavos et al. noted an association between diabetes and low-grade inflammation in a group of people without diagnosed CVD [46]. A study by Okdahl et al. pointed out that inflammation associated with glycemic disorders may contribute to the development of adverse comorbidities [47]. Increasingly, it is considered that effective therapy for cardiometabolic conditions should be based on mechanisms linking inflammation and diabetes [48].

It is also worth mentioning the new PHR index proposed by Jialal et al. [49]. In our study, we noted higher PHR levels in the subgroup with elevated glucose levels (*p* = 0.041) and in the subgroup with higher BMI (*p* = 0.001) compared to the subgroups with lower glucose and BMI, respectively. Jialal et al. reported increased values of this parameter in patients with developing metabolic syndrome. Preliminary reports on PHR indicate that it may be an important marker not only of metabolic syndrome but also of cardiovascular risk and future thrombotic incidents [49]. The combination of platelet count and HDL-C is a novel approach and needs to be confirmed in further studies, but it has been suggested that it may be a better prognostic indicator than platelet count and MPV, with significant value in identifying individuals with increased cardiovascular risk [49].

Moreover, in our study on healthy subjects, we found a statistically significant negative correlation between platelet count and MPV, as well as between MPV and cardiometabolic parameters such as LDL-C or non-HDL-C. The MPV parameter reflects platelet size and is considered to be an indicator that informs us of platelet function and activation [50]. However, a study by Noris et al. noted the relatively small differences in MPV between controls and patients, as well as the fact that variation in MPV can be highly influenced by platelet count, so this parameter should not play a key role in determining the risk and prognosis of various conditions [51]. In a study by Bessmann et al., similar to our study, the MPV was negatively correlated with platelet count in a healthy population [52]. Moreover, a study by Biswaroop et al. of the association between lipid profile and MPV indicated a negative correlation between MPV and TC and LDL-C, although the differences were not statistically significant [53]. In contrast, a study by Khan et al. reported a statistically significant negative correlation between TC and MPV (*r* = −0.088, *p* = 0.011). Higher MPV values were noted only among those with extremely high TC levels (*p* = 0.039) [54]. Moreover, a study by Kim et al. reported a negative correlation between MPV and glucose among people without impaired glycemia [55]. Importantly, there are still relatively limited studies involving individuals with abnormal levels of particular cardiovascular parameters, but still without diagnosed cardiovascular disorders. Thus, the correlation between MPV and platelet count, glucose, or cholesterol remains controversial and inconclusive.

A similar relationship occurs for the inflammatory index, MPVLR, which includes MPV and leukocytes. In our study, we noted a statistically significant negative correlation between both MPVLR and platelet count, and MPVLR and TC, LDL-C, non-HDL-C, and TG. In advanced disease conditions, when there is a change in platelet parameters, MPV or MPVLR may be meaningful. However, it seems that among healthy individuals with platelet parameters within reference ranges and without inflammation, or those with abnormal levels of a certain cardiometabolic parameter but without serious disorders developed, these parameters are not a good source of information about platelets or inflammation.

The main intent behind our study was to attempt to identify platelet-related parameters that could supplement the traditional panel of laboratory tests used to screen people at increased cardiovascular risk and support primary prevention of CVD, as well as reduce the upward tendency of developing adverse chronic cardiometabolic disorders.

The optical aggregometry method is a reliable method for assessing platelet function. It can also support the identification of individuals with a prothrombotic tendency, due to platelet hyperreactivity, and thus an increased probability of developing CVD. Our work follows the approach of Cofer et al. Future research on platelet function should lead to the identification of a population at risk for CVD in which prophylactic use of acetylsalicylic acid would provide notable benefits, and this approach will be a new component of targeted antiplatelet therapy for primary prevention of CVD [56].

Bearing in mind our results from preliminary research, which suggest an association between cholesterol and platelet reactivity, as well as oxidative stress, we point an interesting direction for further research on platelet function in hyperlipidemia. Furthermore, the relationship noted in our study between TG, glucose, BMI, and the relatively new inflammatory markers (MII-1 and MII-3) suggests considering their introduction into clinical practice after further research. These platelet-based inflammatory markers are simple indicators, calculated based on the complete blood count and the traditional inflammatory parameter, CRP, without any additional cost. In summary, an innovative aspect of our study was the inclusion of a group of potentially healthy donors, among whom, as it appeared during the study, some individuals had elevated levels of cardiometabolic parameters. According to the literature, there are studies that consider inflammatory markers or platelet function among patients with already advanced cardiovascular disorders. The intention of our study was to attempt to select platelet-related indicators that may be associated with increased cardiovascular risk among the healthy population. An interesting observation from this study is the significant association between indices calculated from complete blood count and those associated with CRP with traditional risk factors. We believe that platelets may be considered as a non-traditional CVD risk factor.

This study may provide the basis for a prospective cohort study that may confirm that platelets should be considered as a non-traditional CVD risk factor and a key element in identifying individuals at high-risk of developing CVD in the future. This study also contributes to the discussion on screening methods for individuals requiring primary prophylaxis with acetylsalicylic acid [56]. Moreover, the combination of cardiovascular risk factors and platelet-related markers may allow for the prediction of better antiplatelet drug efficacy.

### Study Limitation

Some limitations of our study should be pointed out. We focused on clinical CVD risk factors such as elevated glucose levels, abnormal lipid profile, and excessive body weight. We should acknowledge that our study may not have considered all potential confounding factors. Factors such as genetics (family history of CVD), lifestyle choices (diet, physical activity, alcohol consumption, smoking), and other medical conditions may influence both platelet-related parameters and CVD risk. Future research should aim to control for these variables. In light of these limitations, our study highlighted the need for more longitudinal and comprehensive investigations, as these would provide a better understanding of the potential long-term cardiovascular disease risk associated with platelet-related parameters in individuals who appear to be healthy but may be at risk of developing CVD in the future. We suggest that future research endeavors consider this aspect to enhance the depth of knowledge in this area.

## 4. Materials and Methods

### 4.1. Chemicals

Collagen and thrombin were purchased from Chrono-log Co (Havertown, PA, USA). Antibodies for flow cytometry (anti-human CD62/PE, CD61/PE, CD61/FITC), 0.105 M buffered sodium citrate or acid-citrate-dextrose (ACD), and CellFix were from Becton-Dickinson (Franklin Lakes, NJ, USA). Fibrinogen from Human Plasma Oregon Green 488 Conjugate was received from Invitrogen (Carlsbad, CA, USA). Prostaglandin E1, bovine serum albumin (BSA), dimethyl sulfoxide (DMSO), and dihydroethidium (DHE) were provided by Sigma (St. Louis, MO, USA). Phosphate buffered saline (PBS) was delivered by Corning (New York, NY, USA). All other chemicals, unless otherwise stated, were supplied by Avantor Performance Materials Poland S.A. (Gliwice, Poland).

### 4.2. Study Population

Forty-nine women and forty-five men aged 20 to 71, recruited at the Department of Haemostasis and Haemostatic Disorders (Medical University of Lodz), participated in this study. According to the exclusion and inclusion criteria, donors were included in the study if they had not taken any anticoagulant or antiplatelet drugs for at least fourteen days prior to the study. Volunteers were excluded from the study if, during recruitment, they had elevated levels of C-reactive protein (>10 mg/dL), had chronic inflammatory disease, had infectious disease, or had a cancer diagnosis. All the potential blood donors were introduced to the purpose and procedure of the study and qualified for participation after giving written, voluntary, and informed consent. The research was carried out following approval by the Bioethical Commission at the Medical University of Lodz (RNN/153/20/KE).

### 4.3. Blood Collection and Preparation

Blood was collected fasting in the morning between 8 a.m. and 9 a.m. in a single donation (maximal volume of 30 mL) into polypropylene tubes (S-Monovette^®^). 0.105 M buffered sodium citrate solution (blood/anticoagulant = 9:1 *v*/*v*) was added to the tubes intended for assays with platelet-rich plasma (PRP), or acid citrate dextrose (blood/anticoagulant = 6:1 *v*/*v*) was added to the tubes for assays with isolated platelets. To ensure equal mixing of blood and anticoagulant, the tubes were gently inverted immediately after blood collection. Next, the whole blood was centrifuged to obtain PRP (12 min, 190× *g*, 37 °C). The remaining fraction after supernatant (PRP) removal was centrifuged (15 min, 800× *g*, 37 °C) to receive platelet-poor plasma (PPP). Prostaglandin E1 (50 ng/mL) was added to the tubes with whole blood and PRP, which was centrifugated (15 min, 800× *g*, 37 °C) to obtain isolated platelets. The platelet sediment was then resuspended in Tyrode’s buffer (pH 7.4; 134 mM NaCl, 2.9 mM KCl, 12 mM NaHCO_3_, 0.34 mM Na_2_HPO_4_, 1 mM MgCl_2_, 10 mM HEPES, 5 mM glucose) containing 0.2% bovine serum albumin (BSA). An automatic hematology analyzer Sysmex XS-800i™ (Sysmex, Kobe, Japan) was used to measure the platelet count. Platelets diluted with Tyrode’s buffer to 4 × 10^8^ cells/mL were used to generate reactive oxygen species in isolated platelets, and to 2 × 10^8^ cells/mL for optical aggregation and flow cytometry measurements (fibrinogen binding to platelets and P-selectin expression).

### 4.4. Blood Morphology and Biochemical Parameters from Laboratory Results

Based on laboratory analysis (cooperation with the hospital laboratory at the Medical University of Lodz), each participant received information on blood morphology (platelet count (PLT), mean platelet volume (MPV), platelet crit (PCT), platelet distribution width (PDW), white blood cells (WBC), neutrophils (NEU), lymphocytes (LYM), monocytes (MON), eosinophils (EOS), basophils (BAS), red blood cells (RBC), hemoglobin (HGB), hematocrit (HCT), mean corpuscular volume (MCV), mean corpuscular hemoglobin (MCH), mean corpuscular hemoglobin concentration (MCHC), red cell distribution width (RDW)), and biochemical parameters (including a lipid profile, glucose, uric acid, creatinine, glomerular filtration rate (GFR), bilirubin, aspartate aminotransferase, alanine aminotransferase, and C-reactive protein).

### 4.5. Inflammatory Parameters

Parameters from complete blood count and C-reactive protein (CRP) were used to calculate inflammatory rates: PLR (platelet/lymphocyte ratio); MPVLR (mean platelet volume/lymphocyte ratio); SII—systemic immune-inflammatory index (neutrophil × platelet/lymphocyte ratio); AISI—aggregate index of systemic inflammation (neutrophil × platelet × monocyte/lymphocyte ratio); MII-1—multi-inflammatory index-1 (PLR × CRP); and MII-3—multi-inflammatory index-3 (SII × CRP).

### 4.6. Platelet Aggregation in Platelet-Rich Plasma

Optical aggregation in PRP was performed to assess platelet reactivity in response to collagen. Whole blood was centrifuged to receive PRP (12 min, 190× *g*, 37 °C) and then centrifuged again to obtain PPP (15 min, 800× *g*, 37 °C), used as a blank. Platelet aggregation was induced with collagen (1 µg/mL). Aggregation was monitored for 10 min in PRP samples (200 × 10^6^ platelets/mL) at rest (0–10 min) and after 1 h at 37 °C using an optical aggregometer (Chrono-Log 490-4D, Havertown, PA, USA). Platelet reactivity was assessed based on maximum aggregation value (A_max_) and area under the curve (AUC).

### 4.7. Generation of Reactive Oxygen Species in Isolated Platelets

This part of the experiment was designed to assess the generation of reactive oxygen species (ROS), mainly superoxide anion (O_2_^•−^), in platelets stimulated with thrombin and collagen. To detect O_2_^•−^, dihydroethidium (DHE), a fluorescent probe for intracellular ROS detection, was used. DHE is cell-permeable and upon oxidation by O_2_^•−^, forms a red fluorescent compound, 2-hydroxyethidium (2-OH-E+). 2-OH-E+, thanks to its cationic nature, is able to bind with platelets. Platelets were isolated from whole blood via differential blood centrifugation, initially into PRP (12 min, 190× g, 37 °C), and then into platelet sediment (15 min, 800× g, 37 °C), which was then resuspended in Tyrode’s buffer. After that, platelets (4 × 10^8^ cells/mL) were incubated with 10 µM DHE for 15 min and next were stimulated with collagen (10 µg/mL) or thrombin (0.1 U/mL) for 5 and 10 min. All the samples were diluted 10-fold in Tyrode’s buffer prior to the measurements. The level of ROS generated was assessed via the intensity of the fluorescence emitted by the fluorochromes. The measurements were carried out with a FACSCanto II flow cytometer (Becton Dickinson, Franklin Lakes, NJ, USA). A total of 10,000 cells were collected, and the results were shown as a percentage of marker-positive platelets. 2-OH-E+ in pH 7.4 has an excitation of 520 nm and an emission of 610 nm. During measurements, a 488 nm laser and PE fluorescence filter (585/42 nm) were used. It should be noted that this setting does not filter out the fluorescence of ethidium (E+), which can be formed during a reaction between DHE and other ROS.

### 4.8. Platelet Activation and Reactivity in Platelet-Rich Plasma

Flow cytometry measurements of fibrinogen-binding capacity to platelets and P-selectin (CD62) expression on platelets were performed to assess platelet activation and reactivity. The following antibodies were used: anti-CD61 (FITC-labeled) and anti-CD62 (PE-labeled). The measurements were carried out in PRP samples (200 × 10^6^ platelets/mL) without an agonist (activation) and in samples stimulated with collagen (reactivity) for 5 min, at two incubation times: in platelets at rest (0–10 min) and in platelets after 1 h at 37 °C.

#### 4.8.1. Fibrinogen Binding to Platelets

PRP samples containing exogenous fibrinogen (labeled with 3 µg/mL Oregon Green) were activated with collagen (10 µg/mL) for 5 min at room temperature (RT), and after that, diluted with phosphate-buffered saline (PBS). Then samples were labeled with the appropriate antibodies (anti-CD61/PE for platelet gating) for 15 min at RT and fixed with CellFix (30 min at 37 °C or 60 min at RT). PBS was used to dilute the samples prior to measurements. The conformational change of the glycoprotein IIb/IIIA complex, which occurs as a result of platelet activation, causes the exposure of the fibrinogen binding site, and allows platelets to bind fibrinogen. The assessment of the fraction of activated platelets was carried out with a FACSCanto II flow cytometer (Becton Dickinson, Franklin Lakes, NJ, USA). A total of 10,000 cells (CD61/PE-positive subjects) were collected, and the results were shown as a percentage of marker-positive platelets.

#### 4.8.2. P-Selectin (CD62) Exposure

PRP samples were activated with collagen (10 µg/mL) for 5 min at RT and then labeled with the appropriate antibodies (anti-CD61/FITC for platelet gating and anti-CD62/PE for detection of activated platelets) for 15 min at RT, after which samples were fixed with CellFix (30 min at 37 °C or 60 min at RT). PBS was used to dilute the samples prior to measurements. The assessment of the surface expression of P-selectin (CD62P), as the fraction of activated platelets, was carried out with a FACSCanto II flow cytometer (Becton Dickinson, Franklin Lakes, NJ, USA). A total of 10,000 cells (CD61/FITC-positive subjects) were collected, and the results were shown as a percentage of marker-positive platelets.

### 4.9. Measurement of CD40 Ligand and Interleukin-6 (IL-6) Levels in Plasma

Human plasma, which was stored at -80 °C, was used to determine the level of interleukin-6 (IL-6) and CD40 ligand (CD40L). Measurements were carried out using commercially available enzyme-linked immunosorbent assay (ELISA) Kits (R&D Systems, Minneapolis, MN, USA), following the manufacturer’s protocol. For IL-6, the detection range was 3.1–300 pg/mL (sensitivity 0.7 pg/mL), while for CD40L, the detection range was 62.5–4000 pg/mL (sensitivity 10.1 pg/mL). Measurements were performed with a Victor Microplate Reader (PerkinElmer, Waltham, MA, USA), and absorbance was measured at 450 nm. Results were expressed as pg/mL.

### 4.10. Statistical Analysis

Statistica v.13 (Dell Inc., Tulsa, OH, USA) was used to perform statistical analysis. The normality of the distribution of the analyzed variables was assessed by the Shapiro–Wilk test. Results were presented as mean ± SD (normal distribution) or as median and interquartile range (IQR) (non-normal distribution). The statistical significance of differences between the two groups was assessed using the unpaired Student’s *t*-test (normal distribution) or the Mann–Whitney U-test (non-normal distribution). Pearson’s correlation coefficient or Spearman’s rank correlation was applied to assess the associations between the analyzed parameters for variables with normal or non-normal distributions, respectively. For the multiple regression analysis, a Box–Cox transformation for variables without normal distribution was performed. Multiple regression analysis was performed to evaluate the relationship between platelet-related parameters and cardiometabolic parameters. Statistical significance was established at the *p* value < 0.05.

## 5. Conclusions

In conclusion, our results shed new light on the consideration of platelets as a non-traditional cardiovascular risk factor. To our knowledge, this is the first study with a multi-faced analysis of such different parameters related to platelets in relation to cardiometabolic risk factors performed among theoretically healthy donors. In our study, we highlighted the association between TC and LDL-C with platelet aggregation and platelet ROS generation, as well as the prevalence of elevated levels of inflammatory markers related to platelets MII-1 and MII-3 among people with increased levels of TG, glucose, and excessive body weight. Moreover, PHR is a relatively new and noteworthy parameter requiring further study, but with promising prognostic value in CVD. Interestingly, MPVLR in healthy individuals with abnormal levels of cardiometabolic parameters but without advanced disease is not a good source of information about platelets and inflammation. Undeniably, further studies on platelets and cardiometabolic parameters are needed, not only among patients but especially among healthy people without the normal range of cardiometabolic parameters who will thus be exposed to cardiovascular risk in the future.

## Figures and Tables

**Table 1:** The basic characteristics of the study group (*n* = 94), including selected platelet and biochemical parameters, inflammatory markers, and platelet functions indices.

Variable	All Donors (*n* = 94)
Age (years)	36.0 (26.0–52.0)
BMI (kg/m^2^)	24.7 ± 3.7
PLT (×10^3^/µL)	248 (211–286)
MPV (fL)	10.4 (9.9–11.0)
TC (mmol/L)	5.25 ± 1.04
LDL-C (mmol/L)	3.21 ± 0.91
HDL-C (mmol/L)	1.51 (1.29–1.73)
non-HDL-C (mmol/L)	3.72 ± 1.05
TG (mmol/L)	1.12 (0.81–1.43)
Glucose (mmol/L)	5.27 ± 0.43
CRP (mg/L)	1.2 (0.6–1.9)
PLR	118.5 (101.9–149.0)
SII	371.8 (273.2–496.0)
MII-1	140.4 (79.3–243.0)
MII-3	454.5 (195.9–738.6)
PHR	171.0 ± 47.0
MPVLR	5.1 (4.4–6.3)
Platelet aggregation at rest (collagen 1 µg/mL), A_max_ (%)	83.5 (79.3–86.8)
Platelet aggregation after 1 h at 37 °C (collagen 1 µg/mL), A_max_ (%)	80.1 (73.1–84.5)
Platelet ROS generation, 5 min (collagen 10 µg/mL) (%)	5.2 (3.7–7.6)
Platelet ROS generation, 10 min (collagen 10 µg/mL) (%)	6.5 ± 2.6

Variables were presented as raw values as mean ± SD (normal distribution) or median (IQR) (non-normal distribution). BMI—body mass index; PLT—platelet count; MPV—mean platelet volume; TC—total cholesterol; LDL-C—low-density lipoprotein cholesterol; HDL-C—high-density lipoprotein cholesterol; non-HDL-C—non-high-density lipoprotein cholesterol; TG—triglycerides; CRP—C-reactive protein; PLR—platelet/lymphocyte ratio; SII—systemic immune-inflammation index (neutrophil × platelet/lymphocyte); MII-1—multi-inflammatory index-1 (PLR × CRP); MII-3—multi-inflammatory index-3 (SII × CRP); PHR—platelet/HDL-C ratio; MPVLR—MPV/lymphocyte ratio; A_max_—maximal aggregation; ROS—reactive oxygen species.

**Table 2:** Characteristics of platelet count, platelet aggregation, and platelet reactive oxygen species generation in relation to total cholesterol and LDL cholesterol levels.

Parameter	PLT (×10^3^/µL)	Optical Aggregation (PLT at Rest)	Optical Aggregation (PLT after 1 h at 37 °C)	ROS Generation (Collagen, 5 min)	ROS Generation (Collagen, 10 min)
TC (cut-off: 5.23 mmol/L)					
lower TC	249 ± 53	81.5 (76.9–87.0)	77.5 (69.0–83.3)	4.5 (3.2–6.8)	5.9 ± 2.4
higher TC	257 ± 54	83.8 (81.5–86.8)	81.3 (77.5–84.5)	6.1 (4.6–7.9)	7.1 ± 2.8
*p* Value	0.483	0.163	0.040	0.031	0.040
LDL-C (cut-off: 3.13 mmol/L)					
lower LDL-C	239 (209–268)	81.8 (76.5–87.8)	77.1 (69.0–82.8)	4.6 (3.6–7.6)	6.3 ± 2.8
higher LDL-C	262 (218–296)	83.8 (80.9–86.1)	81.9 (77.8–84.8)	5.9 (4.3–7.5)	6.8 ± 2.5
*p* Value	0.026	0.229	0.022	0.302	0.424

Results were presented as mean ± SD (normal distribution) or median (IQR) (non-normal distribution). Student’s *t*-test (normal distribution) or Mann–Whitney U test (non-normal distribution) were used to assess the significance of differences between two groups (lower vs. higher TC; lower vs. higher LDL-C). PLT—platelet count; ROS—reactive oxygen species; TC—total cholesterol; LDL-C—low density-lipoprotein cholesterol.

**Table 3:** Characteristics of platelet count, platelet to HDL cholesterol ratio (PHR), and multi-inflammatory indexes (MII-1 and MII-3) in relation to triglycerides, glucose, and body mass index levels.

Parameter	PLT (×10^3^/µL)	PHR	MII-1	MII-3
TG (cut-off: 1.12 mmol/L)				
lower TG	249 ± 50	164.0 ± 46.8	108.0 (52.2–192.0)	329.5 (134.4–615.5)
higher TG	257 ± 56	178.0 ± 46.7	192.2 (100.8–276.7)	597.0 (357.3–1030.6)
*p* Value	0.448	0.149	0.002	0.001
Glucose (cut-off: 5.25 mmol/L)				
lower glucose	238 (210–273)	161.1 ± 46.9	88.9 (52.2–181.6)	288.5 (134.4–597.0)
higher glucose	260 (222–298)	180.9 ± 45.5	192.2 (134.0–271.6)	629.4 (409.3–890.1)
*p* Value	0.047	0.041	<0.001	<0.001
BMI (cut-off: 24.3 kg/m^2^)				
lower BMI	231 (202–259)	147.2 ± 38.9	92.5 (50.5–178.9)	280.3 (114.4–506.7)
higher BMI	258 (211–296)	181.7 ± 46.8	189.4 (81.0–269.3)	557.3 (218.9–744.6)
*p* Value	0.059	0.001	0.005	0.002

Results were presented as mean ± SD (normal distribution) or median (IQR) (non-normal distribution). Student’s *t*-test (normal distribution) or Mann–Whitney U test (non-normal distribution) were used to assess the significance of differences between two groups (lower vs. higher TG; lower vs. higher glucose; lower vs. higher BMI). PLT—platelet count; PHR—platelet/HDL cholesterol ratio; MII-1—multi-inflammatory index-1 (PLR × CRP); MII-3—multi-inflammatory index-3 (SII × CRP); TG—triglycerides; BMI—body mass index.

**Table 4:** Multiple regression analysis assessing total cholesterol and LDL cholesterol levels as dependent variables.

	TC (mmol/L)			LDL-C (mmol/L)		
	β	SE	*p* Value	β	SE	*p* Value
PLT	0.169	0.103	0.103	0.184	0.103	0.078
PLT ^A^	0.151	0.098	0.129	0.168	0.100	0.095
PLT ^B^	0.242	0.100	0.017	0.274	0.096	0.005
PLT ^C^	0.220	0.096	0.024	0.255	0.093	0.008
AG_0	0.181	0.109	0.099	0.159	0.110	0.152
AG_0 ^A^	0.244	0.104	0.021	0.228	0.106	0.035
AG_0 ^B^	0.216	0.107	0.047	0.197	0.106	0.066
AG_0 ^C^	0.279	0.102	0.008	0.264	0.102	0.011
AG_60	0.217	0.111	0.055	0.185	0.113	0.106
AG_60 ^A^	0.254	0.106	0.019	0.223	0.108	0.043
AG_60 ^B^	0.247	0.109	0.027	0.218	0.109	0.049
AG_60 ^C^	0.282	0.104	0.008	0.253	0.104	0.018
ROS_5	0.178	0.116	0.129	0.203	0.116	0.085
ROS_5 ^A^	0.159	0.113	0.164	0.186	0.115	0.110
ROS_5 ^B^	0.149	0.116	0.204	0.166	0.115	0.151
ROS_5 ^C^	0.128	0.113	0.263	0.147	0.113	0.197
ROS_10	0.169	0.116	0.150	0.183	0.117	0.120
ROS_10 ^A^	0.148	0.113	0.196	0.164	0.115	0.160
ROS_10 ^B^	0.139	0.117	0.237	0.145	0.115	0.211
ROS_10 ^C^	0.115	0.114	0.314	0.124	0.113	0.280

TC—total cholesterol; LDL-C—low-density lipoprotein cholesterol; PLT—platelet count; AG_0—collagen-induced aggregation in platelets at rest; AG_60—collagen-induced aggregation in platelets after 60 min at 37 °C; ROS_5—reactive oxygen species (ROS) generation in platelets stimulated with collagen for 5 min; ROS_10—ROS generation in platelets stimulated with collagen for 10 min; β—regression coefficient; SE—standard error of the β coefficient; ^A^—model adjusted for age; ^B^—model adjusted for gender; ^C^—model adjusted for age and gender.

**Table 5:** Multiple regression analysis assessing triglycerides, glucose, and body mass index levels as dependent variables.

	TG (mmol/L)			Glucose (mmol/L)			BMI (kg/m^2^)		
	β	SE	*p* Value	β	SE	*p* Value	β	SE	*p* Value
PLT	0.101	0.104	0.333	0.129	0.103	0.214	0.217	0.118	0.069
PLT ^A^	0.086	0.101	0.398	0.115	0.101	0.259	0.194	0.109	0.080
PLT ^B^	0.174	0.101	0.087	0.198	0.101	0.053	0.367	0.103	0.001
PLT ^C^	0.157	0.099	0.116	0.181	0.099	0.072	0.336	0.095	0.001
PHR	0.288	0.100	0.005	0.251	0.101	0.015	0.452	0.107	<0.001
PHR ^A^	0.274	0.098	0.006	0.237	0.099	0.019	0.426	0.099	<0.001
PHR ^B^	0.250	0.097	0.012	0.217	0.099	0.032	0.430	0.094	<0.001
PHR ^C^	0.238	0.095	0.014	0.205	0.097	0.038	0.409	0.087	<0.001
MII-1	0.356	0.098	<0.001	0.372	0.097	<0.001	0.372	0.112	0.001
MII-1 ^A^	0.334	0.096	0.001	0.351	0.096	<0.001	0.365	0.102	0.001
MII-1 ^B^	0.340	0.094	<0.001	0.358	0.094	<0.001	0.357	0.099	0.001
MII-1 ^C^	0.320	0.092	0.001	0.338	0.093	<0.001	0.352	0.090	<0.001
MII-3	0.405	0.096	<0.001	0.347	0.098	0.001	0.370	0.112	0.002
MII-3 ^A^	0.377	0.095	<0.001	0.320	0.098	0.002	0.350	0.103	0.001
MII-3 ^B^	0.382	0.092	<0.001	0.326	0.096	0.001	0.341	0.100	0.001
MII-3 ^C^	0.356	0.092	<0.001	0.301	0.095	0.002	0.325	0.092	0.001

TG—triglycerides; BMI—body mass index; PLT—platelet count; PHR—PLT/HDL cholesterol ratio; MII-1—multi-inflammatory index-1; MII-3—multi-inflammatory index-3; β—regression coefficient; SE—standard error of the β coefficient; ^A^—model adjusted for age; ^B^—model adjusted for gender; ^C^—model adjusted for age and gender.

**Table 6:** The association between MPV or MPVLR and platelet count or the selected cardiometabolic factors.

	MPV (fL)		MPVLR	
	*R_S_*	*p* Value	*R_S_*	*p* Value
PLT (×10^3^/µL)	−0.525	<0.001	−0.303	0.003
TC (mmol/L)	−0.152	0.145	−0.322	0.002
LDL-C (mmol/L)	−0.206	0.048	−0.346	0.001
non-HDL (mmol/L)	−0.226	0.029	−0.348	0.001
TG (mmol/L)	−0.057	0.588	−0.313	0.002
Glucose (mmol/L)	−0.186	0.072	−0.111	0.287
BMI (kg/m^2^)	−0.145	0.229	−0.127	0.290

MPV—mean platelet volume; MPVLR—MPV/lymphocyte ratio; PLT—platelet count; TC—total cholesterol; LDL-C—low-density lipoprotein cholesterol; non-HDL-C—non-high-density lipoprotein cholesterol; TG—triglycerides; BMI—body mass index; *R_S_*—Spearman’s ranks correlation.

## Data Availability

The data presented in this study are available on request from the corresponding author. The data are not publicly available in order to protect patients’ privacy.

## Supplementary Materials

The following supporting information can be downloaded at: https://www.mdpi.com/article/10.3390/ijms241914914/s1.
