# Supplementary material for: Platelets as Potential Non-Traditional Cardiovascular Risk Factor—Analysis Performed in Healthy Donors
Source: Int J Mol Sci. 2023 Oct 5;24(19):14914. doi: 10.3390/ijms241914914 (PMC10573668; doi:10.3390/ijms241914914)
Supplement: Supplementary file 1 [file ijms-24-14914-s001.zip › ijms-2590695-supplementary.pdf]

Platelets as Potential Non-Traditional Cardiovascular Risk Factor – Analysis Performed in Healthy Donors

Patrycja Szymańska [1]*, Bogusława Luzak [1], Przemysław Siarkiewicz [2] and Jacek Golański [1]

Table S1. The basic characteristics of study group (n = 94) including other blood morphology, and biochemical parameters, inflammatory markers, and platelet functions indices.

Variable	All Donors (n = 94)
PCT (%)	0.25 (0.23-0.29)
PDW (fL)	11.7 (10.9-13.3)
PMI (fL/mL)	2.5 (2.3-2.9)
WBC (×10^3/μL)	6.10 ± 1.40
NEU (×10^3/μL)	3.10 (2.54-3.87)
LYM (×10^3/μL)	2.02 (1.70-2.40)
MON (×10^3/μL)	0.53 (0.46-0.62)
EOS (×10^3/μL)	0.16 (0.10-0.22)
BAS (×10^3/μL)	0.04 (0.03-0.05)
RBC (×10^6/μL)	4.78 ± 0.48
HGB (g/dL)	14.4 (13.1-15.5)
HCT (%)	41.7 ± 4.0
MCV (fL)	87 (85-90)
MCH (pg)	30.1 (28.9-30.8)
MCHC (g/dL)	34.2 ± 0.9
RDW (%)	12.7 (12.3-13.1)
Creatinine (μmol/L)	78.7 (66.8-89.6)
GFR (mL/min/1.73m^2)	97.1 ± 17.8
Uric acid (μmol/L)	319.6 ± 86.7
Bilirubin (μmol/L)	12.5 (9.2-15.7)
AST (U/L)	23.1 (19.6-26.7)
ALT (U/L)	19.1 (14.5-33.1)
AISI	190.9 (141.4-275.5)
IL-6 (pg/mL)	3.3 (2.6-4.5)
CD40 (pg/mL)	44.6 (33.2-91.2)
CD62-positive PLT at rest – no agonist (%)	1.8 (1.3-2.3)
CD62-positive PLT after 1 h at 37 °C – no agonist (%)	64.7 (29.8-78.2)
CD62-positive platelets at rest (collagen 10 μg/mL) (%)	2.1 (1.5-3.4)
CD62-positive platelets after 1 h at 37 °C (collagen 10 μg/mL) (%)	67.4 (18.5-80.0)
Fibrinogen-positive PLT at rest – no agonist (%)	3.0 (2.4-4.9)
Fibrinogen-positive PLT after 1 h at 37 °C – no agonist (%)	62.5 (43.4-70.8)
Fibrinogen-positive PLT at rest (collagen 10 μg/mL) (%)	2.3 (1.7-5.0)
Fibrinogen-positive PLT after 1 h at 37 °C (collagen 10 μg/mL) (%)	45.3 ± 21.0
Collagen-induced aggregation – at rest, AUC (%*min)	708.8 (659.8-747.0)

Collagen-induced aggregation – after 1 h at 37 °C, AUC (%*min)	643.8 (538.1-681.6)
Platelet ROS generation, 5 min (thrombin 0.1 U/mL) (%)	9.5 (7.7-12.2)
Platelet ROS generation, 10 min (thrombin 0.1 U/mL) (%)	14.2 ± 4.5

Variables were presented as raw values as mean ± SD (normal distribution) or median (IQR) (non-normal distribution). Student's t-test (normal distribution) or Mann-Whitney U test (non-normal distribution) were used to assess the significance of differences between two groups (females vs. males). PCT – platelet crit; PDW – platelet distribution width; PMI – platelet mass index; WBC – white blood cells; NEU – neutrophils; LYM – lymphocytes; MON – monocytes; EOS – eosinophils; BAS – basophils; RBC – red blood cells; HGB – hemoglobin; HCT – hematocrit; MCV – mean corpuscular volume; MCH – mean corpuscular hemoglobin; MCHC – mean corpuscular hemoglobin concentration; RDW – red cell distribution width; GFR – glomerular filtration rate; AST – aspartate aminotransferase; ALT – alanine aminotransferase; AISI – aggregate index of systemic inflammation (neutrophil × monocyte × platelet/lymphocyte); IL-6 – interleukin-6; CD40 – cluster of differentiation 40; CD62 – P-selectin; PLT – platelets; AUC – area under the curve; ROS – reactive oxygen species.

Table S2. Characteristics of platelet parameters, platelet-based inflammatory markers, IL-6 and CD40 ligand in relation to cardiometabolic risk factors.

Parameter	MPV (fL)	PCT (%)	PDW (fL)	PMI (fL/mL)	PLR	SII	AISI	MPVLR	IL-6 (pg/mL)	CD40 (pg/mL)
TC (cut-off: 5.23 mmol/L)										
lower TC	10.4 (9.9-11.2)	0.25 (0.23-0.28)	11.9 (11.0-13.8)	2.5 (2.3-2.8)	128.1 (106.4-149.0)	362.6 (292.0-511.0)	184.6 (141.4-264.3)	5.3 (4.8-7.2)	3.4 (2.7-4.5)	36.5 (31.1-63.5)
higher TC	10.3 (9.8-10.8)	0.25 (0.22-0.29)	11.6 (10.7-13.3)	2.5 (2.2-2.9)	116.6 (97.4-153.0)	399.5 (270.2-494.3)	203.1 (138.5-276.3)	4.8 (3.9-5.5)	3.2 (2.6-4.6)	50.0 (39.2-137.2)
p Value	0.209	0.982	0.307	0.979	0.104	0.946	0.506	0.002	0.832	0.123
LDL-C (cut-off: 3.13 mmol/L)										
lower LDL-C	10.4 (9.9-11.3)	0.25 (0.22-0.27)	12.0 (11.0-14.2)	2.5 (2.2-2.7)	123.2 (101.2-148.7)	355.8 (253.0-481.6)	179.9 (132.8-264.3)	5.5 (4.6-7.1)	3.4 (2.7-4.5)	36.2 (31.1-54.0)
higher LDL-C	10.3 (9.9-10.8)	0.26 (0.24-0.31)	11.7 (10.7-13.1)	2.6 (2.4-3.0)	118.3 (101.9-153.0)	400.2 (293.1-496.0)	226.5 (151.9-279.0)	4.8 (4.0-5.4)	3.2 (2.6-4.6)	58.6 (43.5-137.2)
p Value	0.192	0.117	0.157	0.109	0.832	0.216	0.141	0.006	0.859	0.035
non-HDL-C (cut-off: 3.74 mmol/L)										
lower non-HDL	10.4 (9.9-11.3)	0.26 (0.23-0.28)	11.9 (11.0-14.3)	2.6 (2.4-2.8)	126.9 (106.6-148.3)	371.6 (292.0-497.2)	183.9 (133.9-265.6)	5.3 (4.8-7.0)	3.4 (2.6-4.5)	38.1 (31.1-62.4)
higher non-HDL	10.3 (9.8-10.8)	0.25 (0.22-0.30)	11.7 (10.7-13.1)	2.5 (2.2-3.0)	116.6 (97.4-155.0)	372.1 (270.2-496.0)	214.5 (146.0-276.3)	4.8 (4.0-5.5)	3.2 (2.6-4.5)	49.7 (39.2-137.2)
p Value	0.252	0.821	0.237	0.806	0.223	0.922	0.315	0.006	0.885	0.138
TG (cut-off: 1.12 mmol/dL)										
lower TG	10.3 (9.9-11.2)	0.25 (0.23-0.28)	11.7 (11.0-13.2)	2.5 (2.3-2.8)	127.5 (104.5-148.3)	358.7 (270.2-511.0)	203.1 (137.8-276.4)	5.4 (4.7-6.8)	3.4 (2.2-4.6)	47.0 (34.8-66.8)
higher TG	10.4 (9.9-11.0)	0.26 (0.22-0.29)	12.0 (10.8-13.3)	2.6 (2.2-2.9)	116.6 (97.4-155.0)	391.4 (279.9-494.3)	183.9 (141.5-272.7)	4.9 (3.9-5.5)	3.3 (2.6-4.5)	41.9 (31.1-108.2)
p Value	0.734	0.573	0.931	0.565	0.263	0.711	0.844	0.024	0.825	0.958
Glucose (cut-off: 5.25 mmol/L)										
lower glucose	10.5 (10.0-11.2)	0.25 (0.22-0.27)	11.9 (11.0-14.0)	2.5 (2.2-2.7)	118.1 (101.4-140.9)	354.2 (253.4-448.2)	184.6 (133.9-264.2)	5.2 (4.1-6.6)	3.2 (2.2-4.3)	44.6 (35.9-91.2)
higher glucose	10.3 (9.7-10.8)	0.26 (0.24-0.31)	11.6 (10.7-13.0)	2.6 (2.4-3.1)	122.7 (102.6-166.3)	418.3 (292.1-536.0)	196.8 (155.3-321.3)	5.1 (4.5-6.2)	3.4 (2.7-4.6)	55.9 (22.4-108.2)
p Value	0.149	0.095	0.250	0.137	0.286	0.061	0.132	0.634	0.169	0.972
BMI (cut-off: 24.3 kg/m²)										
lower BMI	10.5 (10.0-11.6)	0.25 (0.23-0.27)	12.0 (11.0-14.4)	2.5 (2.3-2.7)	118.3 (102.8-131.4)	341.3 (253.0-435.6)	168.2 (132.8-263.1)	5.2 (4.6-6.8)	2.9 (2.1-4.3)	39.7 (31.1-63.5)
higher BMI	10.3 (10.0-10.9)	0.26 (0.23-0.31)	11.7 (11.0-13.2)	2.6 (2.3-3.1)	119.8 (104.5-155.0)	401.0 (286.3-536.0)	238.4 (137.7-276.3)	5.1 (4.4-6.2)	3.4 (2.9-4.6)	53.0 (36.5-108.2)
p Value	0.260	0.320	0.346	0.317	0.331	0.057	0.130	0.337	0.049	0.406

Results were presented as median (IQR) (non-normal distribution). Mann-Whitney U test (non-normal distribution) was used to assess the significance of differences between two groups (lower vs. higher TC; lower vs. higher LDL-C; lower vs. higher non-HDL-C; lower vs. higher TG; lower vs. higher glucose; lower vs. higher BMI). MPV – mean platelet volume; PCT – platelet crit; PDW – platelet distribution width; PMI – platelet mass index; PLR – platelet/lymphocyte ratio; SII – systemic immune-inflammation index (neutrophil × platelets/lymphocyte); AISI – aggregate index of systemic inflammation (neutrophil × monocyte × platelet/lymphocyte); MPVLR – MPV/lymphocyte ratio; IL-6 – interleukin-6; CD40 – cluster of differentiation 40; TC – total cholesterol; LDL-C – low-density lipoprotein cholesterol; non-HDL-C – non-high-density lipoprotein cholesterol; TG – triglyceride; BMI – body mass index.

Table S3. Characteristics of platelet reactivity and platelet ROS generation in relation to cardiometabolic risk factors.

Parameter	Fibrinogen Binding (at Rest)	Fibrinogen Binding (after 1 h at 37 °C)	P-selectin Exposure (at Rest)	P-selectin exposure (after 1 h at 37 °C)	ROS Generation (Thrombin, 5 min)	ROS Generation (Thrombin, 10 min)
TC (cut-off: 5.23 mmol/L)						
lower TC	62.0 (41.3-69.1)	45.7 ± 19.1	60.2 (22.0-74.8)	60.2 (17.3-80.0)	9.4 (7.7-11.5)	13.6 ± 4.3
higher TC	63.0 (47.5-70.9)	44.8 ± 23.1	70.4 (36.3-85.2)	69.8 (21.4-80.6)	9.5 (8.0-12.6)	14.7 ± 4.6
p Value	0.531	0.863	0.127	0.544	0.532	0.255
LDL-C (cut-off: 3.13 mmol/L)						
lower LDL-C	54.9 ± 20.6	45.2 ± 19.9	59.9 (23.1-75.8)	64.9 (17.3-80.0)	9.5 (6.8-12.2)	13.6 ± 4.9
higher LDL-C	58.9 ± 18.1	45.4 ± 22.6	68.6 (45.8-83.3)	72.1 (25.5-80.5)	9.5 (8.1-12.3)	14.8 ± 3.9
p Value	0.408	0.962	0.234	0.559	0.619	0.212
non-HDL-C (cut-off: 3.74 mmol/L)						
lower non-HDL	63.3 (45.8-71.2)	46.5 ± 18.9	64.7 (23.1-75.8)	69.3 (20.5-80.3)	9.8 (7.8-12.2)	14.3 ± 4.6
higher non-HDL	60.4 (41.9-70.8)	43.9 ± 23.5	64.5 (44.0-85.0)	58.5 (14.8-80.0)	9.4 (7.7-12.1)	14.1 ± 4.4
p Value	0.749	0.629	0.424	0.467	0.600	0.870
TG (cut-off: 1.12 mmol/dL)						
lower TG	62.9 (46.9-70.6)	46.1 (35.4-55.2)	64.5 (29.8-78.1)	69.3 (27.3-80.0)	9.5 (7.8-11.5)	14.1 ± 4.7
higher TG	60.5 (41.6-72.0)	46.7 (17.7-62.1)	69.9 (32.8-78.9)	64.9 (14.7-80.4)	9.5 (7.7-12.4)	14.2 ± 4.4
p Value	0.801	0.691	0.791	0.390	0.832	0.932
Glucose (cut-off: 5.25 mmol/L)						
lower glucose	63.6 (46.1-69.6)	52.3 (35.3-65.0)	64.6 (29.8-78.2)	67.9 (20.0-82.1)	9.3 (6.9-12.6)	14.1 ± 5.0
higher glucose	60.3 (41.6-72.4)	41.5 (22.5-53.9)	64.8 (30.6-78.1)	67.1 (16.4-79.1)	9.6 (8.2-11.5)	14.2 ± 3.9
p Value	0.971	0.089	0.860	0.520	0.544	0.879

BMI (cut-off: 24.3 kg/m²)						
lower BMI	60.0 ± 18.1	52.3 (41.2-59.8)	69.0 (48.3-78.1)	77.2 (44.4-83.8)	9.4 (7.8-12.3)	14.3 ± 4.5
higher BMI	56.3 ± 19.0	43.6 (17.9-62.7)	60.2 (23.1-81.5)	36.0 (16.1-75.6)	9.6 (7.7-12.4)	14.7 ± 4.8
p Value	0.464	0.304	0.411	0.014	0.921	0.721

Variables were presented as raw values as mean ± SD (normal distribution) or median (IQR) (non-normal distribution). Student's t-test (normal distribution) or Mann-Whitney U test (non-normal distribution) were used to assess the significance of differences between two groups (lower vs. higher TC; lower vs. higher LDL-C; lower vs. higher non-HDL-C; lower vs. higher TG; lower vs. higher glucose; lower vs. higher BMI). ROS – reactive oxygen species; TC – total cholesterol; LDL-C – low-density lipoprotein cholesterol; non-HDL-C – non-high-density lipoprotein cholesterol; TG – triglyceride; BMI – body mass index.
